# Comment on ‘Systematic Druggable Genome‐Wide Mendelian Randomization Identifies Therapeutic Targets for Sarcopenia’ by Yin et al.—The Author's Reply

**DOI:** 10.1002/jcsm.13652

**Published:** 2024-11-21

**Authors:** Kang‐Fu Yin, Yong‐Ping Chen

**Affiliations:** ^1^ Department of Neurology, West China Hospital Sichuan University Chengdu China

We appreciate the attention and feedback from Liu et al. [1] on our study. We highly value their comments and would like to address some misunderstandings and provide additional background information through the following points.

Firstly, Liu et al. mentioned that colocalisation analysis following Mendelian randomisation (MR) analysis might introduce irrelevant pleiotropic effects by violating the exclusion restriction assumption, thereby not strengthening the MR results. We believe this viewpoint may stem from an incomplete understanding of the principles of two‐sample MR and Bayesian colocalisation analysis. Two‐sample MR extracts strictly valid instrumental variables (IVs), selecting those SNPs that are significantly associated with the exposure variable. These SNPs need to satisfy the exclusion restriction assumption, meaning they influence the outcome variable only through the exposure variable and not through other pathways [2]. The purpose of Bayesian colocalisation analysis is to determine whether a genetic variant simultaneously affects multiple phenotypes. Colocalisation analysis typically identifies SNPs that are significantly associated with multiple phenotypes, i.e., shared SNPs [3]. This means that the IVs selected for MR analysis do not necessarily coincide with the shared SNPs identified by colocalisation, thus avoiding the violation of the exclusion restriction assumption. Even if there is a coincidence, after rigorous IV selection and pleiotropy methods such as MR‐Egger regression and MR‐PRESSO evaluation, the research results remain robust and interpretable [4]. Colocalisation analysis is an important complement to cis‐MR studies, used to evaluate the validity of the IV assumption [4]. The lack of colocalisation analysis in cis‐MR studies may lead to false‐positive results similar to candidate gene studies, which have now largely been abandoned due to their irreproducible findings [5].

Secondly, Liu et al. mentioned irrelevant horizontal pleiotropy and relevant horizontal pleiotropy in their comments. Regarding irrelevant horizontal pleiotropy, we have already employed common MR methods to address pleiotropy in our article, such as MR‐Egger regression and MR‐PRESSO. MR‐Egger regression detects and corrects pleiotropy by estimating the intercept term, while MR‐PRESSO reduces pleiotropic effects by identifying and excluding outliers. However, Liu et al. suggested that pleiotropy might still exist but did not provide specific evidence or reasons. We believe that every methodological approach has its limitations, including the CAUSE (Causal Analysis Using Summary Effect estimates) method suggested by Liu et al. for future pleiotropy identification. Although the CAUSE method performs well in identifying pleiotropy, it also has a high false‐positive rate and cannot fully explain the shared genetic components between two traits of interest [6]. Relevant horizontal pleiotropy refers to a SNP affecting the outcome variable through multiple pathways, which is a common issue in complex trait studies. We agree that this is a topic requiring further research, but it does not undermine the robustness of our current findings [2, 4]. We have employed various methods to detect and correct pleiotropy in our study to ensure the reliability of the results.

Lastly, our study applied cis‐MR analysis and used Bayesian colocalisation results for sensitivity analysis to support our conclusions. This approach has been supported in previous literature [4, 7, 8, 9, 10, 11] and was also recommended in Liu et al.’s comments. Therefore, we believe our results are robust.

Regarding the CAUSE method [6] and the latest TWAS (Transcriptome‐Wide Association Study) [12, 13] algorithms mentioned by Liu et al., these methods may represent future research directions but still require further public validation.

Once again, we thank Liu et al. for their valuable comments. We hope that our responses above can clarify some misunderstandings and look forward to further discussion and exchange.

## Conflicts of Interest

The authors declare no conflicts of interest.
